# Model architectures for bacterial membranes

**DOI:** 10.1007/s12551-021-00913-7

**Published:** 2022-03-07

**Authors:** Ashley B. Carey, Alex Ashenden, Ingo Köper

**Affiliations:** Institute for Nanoscale Science and Technology, College for Science and Engineering, Flinders University, Adelaide, SA 5042 Australia

**Keywords:** Model membrane, Lipids, Membrane, Biophysics, Bacteria

## Abstract

The complex composition of bacterial membranes has a significant impact on the understanding of pathogen function and their development towards antibiotic resistance. In addition to the inherent complexity and biosafety risks of studying biological pathogen membranes, the continual rise of antibiotic resistance and its significant economical and clinical consequences has motivated the development of numerous in vitro model membrane systems with tuneable compositions, geometries, and sizes. Approaches discussed in this review include liposomes, solid-supported bilayers, and computational simulations which have been used to explore various processes including drug-membrane interactions, lipid-protein interactions, host–pathogen interactions, and structure-induced bacterial pathogenesis. The advantages, limitations, and applicable analytical tools of all architectures are summarised with a perspective for future research efforts in architectural improvement and elucidation of resistance development strategies and membrane-targeting antibiotic mechanisms.

**Supplementary Information:**

The online version contains supplementary material available at 10.1007/s12551-021-00913-7.

## Introduction

All organisms rely on the presence of biological membranes acting as barriers between the inside and outside cellular environments. The functionality of such membranes is dictated by the types of lipids and other molecules that make up their often highly complex structure (Watson 2015; Guidotti 1972).

The “ESKAPE” pathogens, a faction of Gram-negative (GN) and Gram-positive (GP) bacteria, are responsible for the majority of nosocomial infections and are deemed a great threat to global healthcare because of their multidrug resistance (MDR) (Boucher et al. 2009; Mar et al. 2017; Pendleton et al. 2013; Rice 2010; Santajit and Indrawattana 2016; Ventola 2015). MDR bacterial pathogens can overexpress intrinsic resistance markers via adaptive mutations and acquire various foreign resistance factors through gene transfer processes (Gould and Bal 2013; Ventola 2015; Chilambi et al. 2018; Fernández and Hancock 2012; Prestinaci et al. 2015; Jiang et al. 2019a). This makes them resistant to even the most effective antimicrobial medications, rendering once treatable infections untreatable (Mar et al. 2017; Renwick et al. 2016). Antimicrobial resistance has resulted in significant economic damage due to increased patient morbidity and mortality (Boucher et al. 2009; Ventola 2015; Renwick et al. 2016; Dutescu and Hillier 2021; D’Andrea et al. 2019; Tacconelli et al. 2018). Given the lack of success in marketing novel therapeutic antimicrobial agents including teixobactins, antimicrobial nanomaterials, and micro-engineered biomolecules (Mulani et al. 2019; Makabenta et al. 2021; Fatima et al. 2021; Mantravadi et al. 2019; Charbonneau et al. 2020; Hussein et al. 2020), current research has been devoted to sourcing natural antimicrobial products due to their chemical diversity and reported effectiveness as narrow- or broad-spectrum antibiotics (Hutchings et al. 2019; Quinto et al. 2019; Ghrairi et al. 2019). However, further research is required to ensure their clinical utility and to develop a better understanding of their mechanism of action. This highlights the critical requirement to understand the mechanisms behind pathogen resistance development and antimicrobial action.

The bacterial lipid membrane of MDR pathogens plays a significant part in the resistance development towards membrane-targeting antibiotics (polymyxins, β-lactams, glycopeptides, and lipopeptides), which typically penetrate the cell membrane to facilitate cellular entry of medication, or directly disrupt the cell membranes structural integrity to facilitate cell lysis (Kapoor et al. 2017; Epand et al. 2016; Tenover 2006; Dias and Rauter 2019). The membrane lipid profile can dictate the effectiveness of antibiotics and drug-efflux proteins that mediate the expulsion of antibiotics from the bacterium. Pathogen adaptation mechanisms alter the native lipid composition which facilitates structural modifications, including changes in membrane fluidity, organisation, and packing, that circumvents the effects of antibiotics and evades host immune attack (Jiang et al. 2019a, 2019b; Dadhich and Kapoor 2020; Han et al. 2018; Maifiah et al. 2016; Mishra et al. 2012). The unique structure of the membrane in GN bacteria is the primary reason for their rapid resistance development compared to GP bacteria (Breijyeh et al. 2020; Ghai and Ghai 2018). The lipid asymmetry, rigidity, and biochemistry of the LPS molecules in the membrane provide a considerable defensive barrier against numerous antibiotics (Breijyeh et al. 2020; Delcour 2009; Vasoo et al. 2015). Changes in the lipophilic composition and membrane structure can also influence various membrane-associated processes such as protein-lipid electrostatic interactions, ligand-binding, cell-to-cell communication, transport, and protein folding, translocation, and function (Corradi et al. 2019; Collinson 2019; Lin and Weibel 2016; Martens et al. 2019, 2016; Norimatsu et al. 2017; Du et al. 2018).

The bacterial lipid membrane is a viable target for novel antibiotic treatments as the lipophilic composition is crucial to antibiotic efficacy, and targeting the lipid membrane rather than biochemical pathways can prolong antibiotic resistance development (Dias and Rauter 2019; Lam et al. 2016). A better understanding of the bacterial lipid membrane and its interactions with antibiotics is thus imperative for subsequent antibiotic research and development efforts.

However, systematic studies of the bacterial cell membrane structure and its processes are difficult to perform when studying live bacterial cells due to the nanometre dimensions of their membranes as well as their high level of complexity (Behuria et al. 2020). Bacteria also possess a cell wall that requires removal prior to investigating membrane-mediated activities (Brown et al. 2010; Veron et al. 2008). The inherent complexity of biological bacterial cell membranes which contain numerous peptides, sugars, membrane proteins, lipids, and carbohydrates makes systematic investigations difficult (Andersson et al. 2018a; Castellana and Cremer 2006). Pathogenic bacteria especially pose unique investigatory challenges due to rigorous biosafety protocols (Behuria et al. 2020). An alternate method to analyse membrane-associated processes is to purify the bacterial membrane; however, the isolation process requires expensive instrumentation which is difficult to perform in common laboratories (Qing et al. 2019). Due to these limitations, progressions in the understanding of the organisation, structure, and processes that occur in biological bacterial membranes have been driven primarily through research on in vitro model membrane systems (Strahl and Errington 2017).

A variety of different model systems have been designed to mimic biological membranes in a controlled environment with only the most essential components (Salehi-Reyhani et al. 2017). Model membranes were developed as an accessible experimental platform to analyse membrane structure and function in an environment that replicates the fundamental environmental and physiochemical properties of biological membranes, whilst reducing their innate complexity (Andersson et al. 2018a,^ 2020^, 2018b; Andersson and Köper 2016; Chan and Boxer 2007; Jackman et al. 2012; Siontorou et al. 2017). Model membrane systems are computationally modelled, free-standing, or solid-supported bilayer structures composed of various lipophilic compounds and proteins (Chan and Boxer 2007; Siontorou et al. 2017).

They enable the use of numerous microscopic, spectroscopic, electrochemical, reflectometric, and algorithmic analytical techniques often inaccessible when studying live cells (Wiebalck et al. 2016; Zieleniecki et al. 2016). The analytical techniques can, for example, reveal the mechanism of action surrounding membrane-targeting antibiotics (Peetla et al. 2009; Knobloch et al. 2015). Numerous model membrane systems have been designed to investigate membrane-drug interactions (Hollmann et al. 2018); however, few mimic bacterial membranes or the architecture of the ESKAPE pathogens.

Here, we provide an overview of the structure and lipophilic composition of GN and GP bacterial membranes and current membrane modelling systems for these structures, including liposomes, solid-supported bilayers, and computational simulations.

## Bacterial membranes

Lipids in bacterial membranes serve as important structural and functional constituents and have important roles in membrane organisation, cell recognition, membrane fluidity, energy storage, direct modulation, membrane stability, cell signalling, and membrane formation (Solntceva et al. 2020; Carvalho and Caramujo 2018; Willdigg and Helmann 2021). To perform such complex and diverse functions, bacterial membranes are composed of approximately equivalent proportions of lipids and proteins and are complex structures with a high degree of organisation and variation between bacterial species and their GN and GP classifications (Strahl and Errington 2017; Epand and Epand 2009a; Sohlenkamp and Geiger 2016).

GN and GP bacterial lipid membranes are predominantly formed by phospholipids which are composed of a phosphate group, 2–4 hydrophobic fatty acid units, a variable hydrophilic head group, and a glycerol moiety (Sohlenkamp and Geiger 2016; Alagumuthu et al. 2019; Fahy et al. 2011). Phospholipids are organised in a classical bilayer described by the fluid-mosaic model (Singer and Nicolson 1972). The model has since been refined to accommodate the presence of lipid domains and cytoskeletal proteins that restrict and sectionalise lipid and protein diffusion (Strahl and Errington 2017; Meer et al. 2008; Barák and Muchová 2013). Both GN and GP bacteria contain a large variety of straight or branched, saturated, or unsaturated carboxylic acids with long aliphatic chains, known as fatty acids, that serve as essential building blocks for multiple lipophilic compounds (Carvalho and Caramujo 2018; Cronan and Thomas 2009). Numerous glycolipids, which are composed of a carbohydrate attached by a glycosidic bond containing 1–2 fatty acid units, are also typical constituents in the membranes of GN and GP bacteria (Bertani and Ruiz 2018; Reichmann and Gründling 2011). In addition to the aforementioned common lipid species, bacteria can also possess species-specific lipids (Solntceva et al. 2020).

Within bacterial species of different and the same Gram types, the lipid membrane contains a high degree of structural, chemical, and functional variability whereby numerous lipid molecular variants are present that differ in size, number, chemical composition, and isomeric form (Strahl and Errington 2017; Sohlenkamp and Geiger 2016; May and Grabowicz 2018; Rahman et al. 2000). Pathogens can also readily acquire multiple exogenous lipophilic bodies which generate substantial variation between pathogen strains and species (Jiang et al. 2019a; Jasim et al. 2018). The key lipid species present in the ESKAPE pathogens has been studied extensively (Table 1) (Sohlenkamp and Geiger 2016).

**Table 1:** Diversity of membrane lipid species documented for the ESKAPE pathogens

Bacterial species	Major membrane lipid species	References
*E. faecium*	PG, CL, Lysyl-PG, GP-DGDAG, Type I LTA, FA	Mishra et al. 2012; Theilacker et al. 2012)
*S. aureus*	PG, CL, Lyso-PG, GPL, Lysyl-PG, Type I LTA, FA	Epand and Epand 2009a; Song et al. 2020; Schneewind and Missiakas 2014; Kilelee et al. 2010; Malanovic and Lohner 2016; Oku et al. 2004; White and Frerman 1967)
*K. pneumoniae*	PG, PE, CL, SL, PC, Lysyl-PG, Lyso-PE, PI, PA, Lyso-PA, Lyso-PC, LPS, FA	Epand and Epand 2009a; Jasim et al. 2018; Vinogradov et al. 2002; Hobby et al. 2019)
*A. baumannii*	PE, PG, CL, Lyso-PE, Acyl-PG, PA, MLCL, PE-OH, CL-OH, MLCL-OH, LPS, FA	Jiang et al. 2019a; Unno et al. 2017; Jiang et al. 2020; Lopalco et al. 2017)
*P. aeruginosa*	PG, CL, PE, PC, OL, Alanyl-PG, RL, LPS, FA	Epand and Epand 2009a; Malanovic and Lohner 2016; Chao et al. 2010; Lam et al. 2011; Klein et al. 2009; Lewenza et al. 2011; Pramanik et al. 1990; Wilderman et al. 2002; Soberón-Chávez et al. 2005)
Enterobacter species^†^ (*E. cloacae*, *E. hormaechei*, *and E. aerogenes)*	PG, PE, CL, LPS, FA	Epand and Epand 2009a; Bøse and Gjerde 1980; Gill and Suisted 1978; Kämpfer et al. 2015; Davin-Regli et al. 2019; Epand and Epand 2009b; Epand et al. 2010)

^†^As there are 22 species found in the *Enterobacter* genus, only common species described in nosocomial infections were analysed and lipid compositions are assumed to be similar between each (same genus)(Davin-Regli et al. 2019; Epand et al. 2010; Villegas and Quinn 2002)

^*^See Supplementary Information (Sects. 1 and 2) for bacterial and lipid species acronym definitions, respectively

**Fig. 1:**
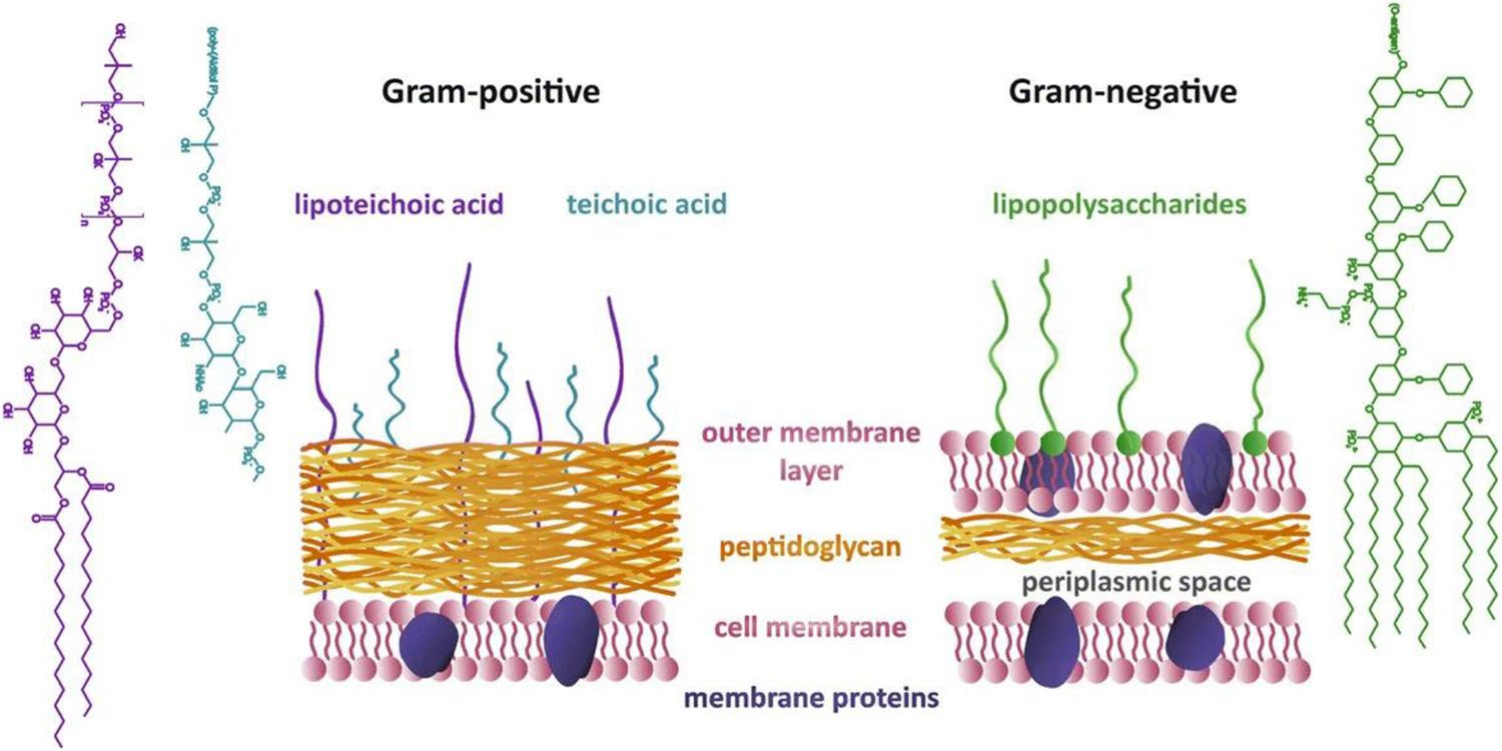
Schematic depiction of the key structural differences in the cell walls of GN and GP bacteria (used with permission from (Pajerski et al. 2019))

GN bacterial membranes consist of two lipid bilayers separated by a viscous, protein-enriched aqueous periplasmic space and a thin peptidoglycan (murein) wall (Fig. 1) (Kapoor et al. 2017; Barák and Muchová 2013; Silhavy et al. 2010). The inner membrane (IM) is comprised of an asymmetric phospholipid bilayer that encases the cytosol and harbours membrane proteins responsible for transport, energy production, protein secretion, and lipid biosynthesis (Silhavy et al. 2010; Bogdanov et al. 2020). The murein wall is responsible for protecting the bacterium against osmotic and mechanical stresses and maintaining bacterium shape (Kapoor et al. 2017; Silhavy et al. 2010). The outer membrane (OM) is attached to the murein wall via lipoproteins (Silhavy et al. 2010). The OM is an asymmetric lipid bilayer surrounding the periplasmic space (Kapoor et al. 2017; Paulowski et al. 2020). The proximal leaflet is comprised of phospholipids, whilst the distal leaflet is predominantly comprised of LPS which functions as a protective barrier (Silhavy et al. 2010; Cian et al. 2020). LPS is a glycolipid constructed of three distinct parts: lipid A (hydrophobic domain), the oligosaccharide core (hydrophilic domain), and the O-antigen (outmost polysaccharide domain) (Raetz and Whitfield 2002; Wang and Quinn 2010). The structure of LPS differs significantly between GN bacterial species due to survival adaptations in response to changes in environmental stimuli including pH, temperature, specific ion concentrations, osmolality, and toxins (including antibiotics) (Li et al. 2012; Needham and Trent 2013; Trent et al. 2006; Simpson and Trent 2019). Biochemical modifications to LPS domains or selective LPS production abandonment (specific to *A. baumannii* only) have been found to allow GN bacterial pathogens to evade host-immune attack, increase pathogenesis, and develop antimicrobial resistance (Needham and Trent 2013; Trent et al. 2006; Simpson and Trent 2019; Maldonado et al. 2016; Moffatt et al. 2010; Pelletier et al. 2013), for example, LPS modification adaptation strategies adopted by GN bacteria to protect themselves from cationic antimicrobials such as polymyxins include hydroxylation, dephosphorylation, palmitoylation, phosphatidylethanolamine addition, and 4-amino-4-deoxy-L-arabinose (L-Ara4N) addition to the lipid A portion (Dortet et al. 2020; Olaitan et al. 2014). The most common and effective modification to LPS in GN bacterial pathogens is the addition of L-Ara4N via cationic substitution of the 4’-phosphate group on the lipid A moiety (Olaitan et al. 2014; Nikaido 2003). This modification reduces the net charge of lipid A which, consequently, decreases the degree of electrostatic repulsion experienced between neighbouring LPS molecules. The incorporation of these cationic constituents results in a net positive charge of LPS upon biosynthesis which, inevitably, repulses cationic antimicrobials (Dortet et al. 2020; Olaitan et al. 2014). This repulsion results in antimicrobial resistance as the membrane has developed protection against OM disruption. In addition, murein lipoproteins and β-barrel proteins are present in the OM for murein wall anchoring and small (anions, maltodextrins, and maltose) and large molecule (antibiotics, vitamins and chelates) diffusion or transport (Silhavy et al. 2010).

The OM and LPS leaflets are absent in most GP bacteria which, in GN bacteria, are crucial in providing an additional stabilising layer around the bacterium and protect the bacterium from environmental hazards (Malanovic and Lohner 2016; Silhavy et al. 2010). To compensate for the OM deficit and withstand the osmotic and mechanical pressures exerted on the plasma membrane, GP bacteria are surrounded by a murein wall that is notably thicker (40–80 nm) in GP bacteria than those found in GN bacteria (7–8 nm) (Kapoor et al. 2017; Epand and Epand 2009a; Barák and Muchová 2013; Malanovic and Lohner 2016; Silhavy et al. 2010). Teichoic acids, including LTA, thread through the murein layers to anchor the murein wall to the membrane and regulate cell envelope function and structure (Malanovic and Lohner 2016; Silhavy et al. 2010). LTA is an alditol phosphate polymer linked by a glycolipid anchor that secures it to the lipid membrane (Solntceva et al. 2020; Percy and Gründling 2014). The structure of LTA varies significantly between GP bacterial species whereby there are five types of LTA (types I–V) that differ in core structure and glycolipid anchor (Percy and Gründling 2014; Shiraishi et al. 2013). Similarly to LPS in GN bacteria, biochemical modifications to the LTA backbone structure have been found to illicit antimicrobial resistance in GP bacterial pathogens (Percy and Gründling 2014; Gutmann et al. 1996; Saar-Dover et al. 2012). For example, the D-alanylation of LTA mediated by the *dlt* operon and/or incorporation of L-lysine in PG via the *mprF* gene can lead to an enhanced resistance against cationic antimicrobials (Percy and Gründling 2014; Saar-Dover et al. 2012; Abachin et al. 2002; Peschel et al. 1999; Reichmann et al. 2013). The modification increases the overall net positive surface charge of the membrane and reduces the binding affinity of cationic antimicrobials (Percy and Gründling 2014; Abachin et al. 2002; Peschel et al. 1999). However, other pathways may also be involved in resistance development. The addition of D-alanine, for example, also changes the conformation of LTA resulting in an increase in cell wall density and cell surface rigidity (Percy and Gründling 2014; Saar-Dover et al. 2012). This leads then to a reduction in the permeation of cationic antimicrobials through the cell. The membranes of GP bacteria are comprised of a single asymmetric phospholipid bilayer that encases the cytosol (Silhavy et al. 2010; Rosado et al. 2015; Jones et al. 2008). As there is no OM in GP bacteria to harbour extracellular proteins, GP bacteria are decorated with numerous proteins bound via peptide anchors, covalent interactions, lipid anchors, or non-covalent interactions to the membrane, murein wall, and/or teichoic acids that perform functions analogous to those found in GN bacteria (Malanovic and Lohner 2016; Silhavy et al. 2010; Scott and Barnett 2006).

## Model membrane systems

Various model membrane systems have been established. Here, we focus on systems that specifically mimic microbial membranes.

### Liposomes

**Fig. 2:**
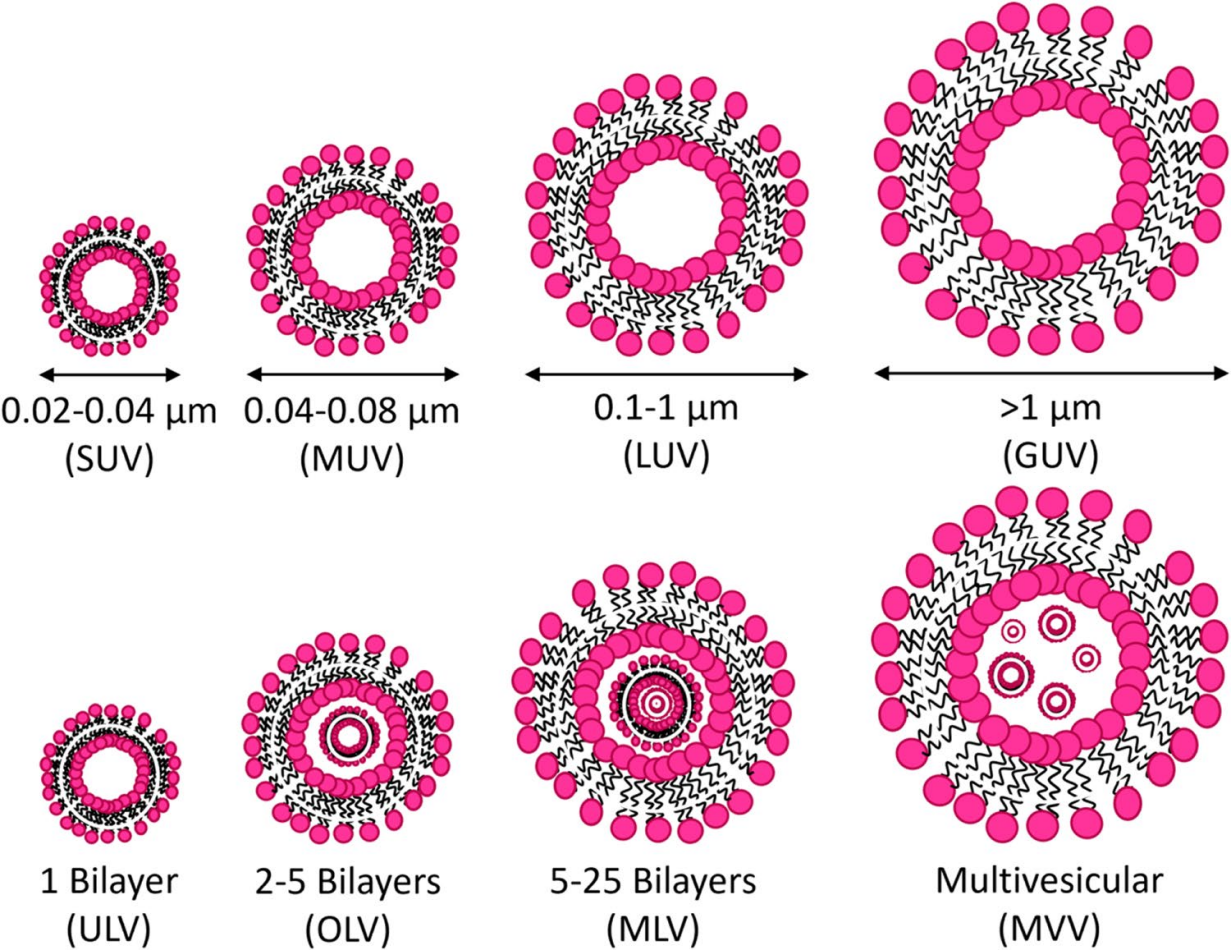
Schematic representation of different sizes (top) and lamellar structures (bottom) of liposomes

Liposomes are spherical-shaped vesicles ranging from nano- to micrometre diameters that are comprised of one or more phospholipid bilayers that encase an aqueous core (Siontorou et al. 2017; Akbarzadeh et al. 2013). Liposome structures are categorised according to their lamellar structure and vesicular size: unilamellar vesicles (ULV) can be small (SUV, 0.02–0.04 µm), medium (MUV, 0.04–0.08 µm), large (LUV, 0.1–1 µm), and giant (GUV, > 1 µm) (Siontorou et al. 2017; Akbarzadeh et al. 2013; Šturm and Poklar Ulrih 2021). Oligolamellar vesicles (OLV) are > 0.5 µm and can contain 2–5 concentrically arranged bilayers, multilamellar vesicles (MLV) are > 0.7 µm and can contain concentrically arranged 5–25 bilayers, and multivesicular vesicles (MVV) are 1–100 µm and can contain one or more non-concentrically arranged internal bilayers (Fig. 2) (Akbarzadeh et al. 2013; Navas et al. 2005; Giuliano et al. 2021; Mu et al. 2018). Liposomes are easily formed via numerous methods as reviewed elsewhere (Siontorou et al. 2017; Akbarzadeh et al. 2013; Šturm and Poklar Ulrih 2021). Liposome properties can differ depending on the method of preparation, size, lipophilic composition, surface charge, and functionalisation which allows for a considerable degree of customisation (Gabizon et al. 1998; Sherratt and Mason 2018; Fan et al. 2007; Bozzuto and Molinari 2015; Riaz et al. 2018; Sakai-Kato et al. 2019).

Liposomes have been constructed to mimic the OM, IM, and cytoplasmic space of various non-pathogenic and pathogenic bacteria (Table 2) (Behuria et al. 2020; Bogdanov et al. 2020; Paulowski et al. 2020; Tuerkova et al. 2020; Dombach et al. 2020; Jamasbi et al. 2014; Kumagai et al. 2019; Pérez-Peinado et al. 2018; Malishev et al. 2018; Kahveci et al. 2016; Lopes et al. 2012; Cheng et al. 2011; Marín-Menéndez et al. 2017; Fernandez et al. 2011; Domenech et al. 2009; Pinheiro et al. 2013; D’Errico et al. 2010; Furusato et al. 2018; Kiss et al. 2021; Jiménez et al. 2011; Sikder et al. 2019; Kubiak et al. 2011; Mohanan et al. 2020; Ruhr and Sahl 1985; Bharatiya et al. 2021).

**Table 2:** Summary of cited liposome models, the lipid source, the lipid species utilised, and their corresponding research outcomes

Model type	Reference	Lipid source	Lipid species	Research outcomes
GUV	Behuria et al. 2020)	*E. coli* polar lipid extract (DH5α)	PE, PG, CL	Development of a facile, inexpensive, and reproducible method for producing bacterial GUVs
	Furusato et al. 2018)	Purchased synthesised lipids	POPC, POPG, Rhod-DOPE	Formation of membrane-associated proteins using a cell-free protein synthesis system inside GUVs
	Jiménez et al. 2011)	*E. coli* lipid extract (JM600)	Unspecified lipid content from the extracts	Incorporation of soluble proto-ring proteins into GUVs for probing of divisome component interactions
	Kubiak et al. 2011)	Purchased *E. coli* B (ATCC 11,303) polar lipid extracts, *E. coli* (O55:B5) LPS extracts, *E. coli* (EH-100) LPS extracts, *E. coli* (J5) LPS extracts, *E. coli* (F583) LPS, and lipid A extracts and synthesised lipids	Extracted: PE, PG, CL, S-LPS, FITC-LPS, Ra-LPS, Rc-LPS, Rd-LPS, MPLA Synthesised: Rhod-DHPE	Development of novel protocol for formation of GUVs composed of LPS species and *E. coli* extracts
	Mohanan et al. 2020)	*E. coli* B (ATCC 11,303) polar lipid extracts and purchased synthesised lipids	Extracted: PE, PG, CL Synthesised: DOPG, Lysyl-PG, TOCL	Development of GUV-based GN and GP bacterial membrane vesicles
	Saliba et al. 2014)	Purchased porcine brain extract, *S. cerevisiae* (yeast) extract, *P. cirerri* (yeast) extract, *and* synthesised lipids	Extracted: PIP, SL (PHS including phosphate forms and phytocer) Synthesised: DAG, POPC, TOCL, DOPS, DOPG, POPE, DOPA, DOPI (including phosphate forms), SL (dihydrocer, cer (including phosphate and fluorescent forms), SO (including phosphate and florescent forms), DHS (including phosphate and fluorescent forms)), PEG350-PE, PEG2000-PE, ATTO647N-DMPE, NBD-PG, bodipy FL-PE Unknown source: ES	Systematic characterisation of protein-lipid interactions using a microarray of liposomes
	Turner et al. 2015)	Purchased synthesised lipids	DOPE, DOPG, TOCL, Lysyl-PG	Analysis of *C. botulinum* toxin type A using culture and liposomal methods to assess loss of sterility
	Paulowski et al. 2020)	LPS extracts from *P. mirabilis* (R_45_). Purchased *E. coli* lipid extracts and synthesised lipids	Extracted: PE, PG, R-LPS Synthesised: CL, Rhod-DHPE, NBD-PE, FITC-PE	Demonstrate experimental methods to model the asymmetry of GN bacteria. The model’s usability was assessed for lipid domain analysis and peptide and protein interaction by characterising lipid flip-flop and phase behaviour
LUV	Sikder et al. 2019)	Purchased synthesised lipids	DPPC, DPPG, DPPE	Programmable supramolecular assembly of π-amphiphile(s) for determination of interactions with bacteria and membrane mimicking liposomes
	Som and Tew 2008)	Purchased *E. coli* B (ATCC 11,303) total lipid extracts and synthesised lipids	Extracted: PE, PG, CL, unspecified lipid content, Egg-Lyso-PC Synthesised: DOPE, DOPC, DOPG, DOPS	Use of a variety of lipid and lipid extract combinations to show that lipid structure and type could be more important than headgroup charge for determining membrane selectivity towards multiple antimicrobial oligomers
	Samuel and Gillmor 2016)	Purchased synthesised lipids	DOPG, DOPC, DPPC, DPPG	Examination of kinetics, behaviours and potential mechanisms of the NA-CATH peptide using SUVs
	Sborgi et al. 2016)	Purchased *E. coli* B (ATCC 11,303) polar lipid extract, porcine brain total lipid extract and synthesised lipids	Extracted: PG, PE, CL, PA, PS, PI, PC, unspecified lipid content Synthesised: DMPC	Determination that gasdermin D is the direct and final executor of pyroptotic cell death using liposome-inserted gasdermin D
	Carrasco-López et al. 2011)	Purchased *E. coli* B (ATCC 11,303) lipid extract (unspecified)	Polar (PE, PG, CL) or total (PE, PG, CL, unspecified lipid content)	Investigate the activation mechanism of AmpD peptidoglycan amidase to represent the regulatory processes that occur for other intracellular members of the amidase_2 family
	Sasaki et al. 2019)	Purchased *E. coli* B (ATCC 11,303) polar lipid extract and synthesised lipids	Extracted: PG, PE, CL Synthesised: DAG	Determination that YidC accelerates MPIase-dependent membrane protein integration
	Cheng et al. 2014)	Purchased synthesised lipids	POPC, POPG, POPE	Mechanistic contributions of membrane depolarisation in *S. aureus* towards the bactericidal activity of ramoplanin
	Lombardi et al. 2017)	Purchased bovine heart CL extract and synthesised lipids	Extracted: CL Synthesised: DOPE, DOPG, DPPE, DPPG, NBD-PE, Rhod-PE, 5-SLPC, 14-SLPC	Perturbation of lipid membranes by myxinidin mutant WMR due to anionic lipid segregation
	Zhang et al. 2014)	Purchased synthesised lipids	DMPC, DMPG, TOCL	Using cardiolipin in liposomes to show that changes in membrane lipid composition can allow bacteria to become resistant to daptomycin
	Domenech et al. 2009)	Purchased bovine heart CL extracts and synthesised lipids	Extracted: CL Synthesised: POPC, DPPG, POPG, POPE	Investigate the effect of vancomycin and oritavancin on the permeability and organisation of phospholipids in bacterial membrane models
	Fernandez et al. 2011)	Purchased synthesised lipids	DMPC, DMPG, d-DPMC, d-DMPG	Investigate the drug-membrane interactions between the synthetic antimicrobial peptide P5 and bacterial and human membrane models using solid-state NMR and circular dichroism
	Marín-Menéndez et al. 2017)	Purchased synthesised lipids	POPC, PG, CL	Develop bacterial model membranes to investigate the drug-membrane interactions and delivery mechanism of oligonucleotide therapeutics
	Lopes et al. 2012)	Purchased *E. coli* B (ATCC 11,303) total lipid extracts	PE, PG, CL, unspecified lipid content	Generate model membranes that represent *Y. kristensenii* and *P. mirabilis* to determine differences in lipid phase transitions with variations in lipid composition ratios
	Jamasbi et al. 2014)	Purchased synthesised lipids	POPE, POPG	Investigate and compare the cytosolic and antimicrobial mechanism of action of the lytic peptide, melittin, between prokaryotic and eukaryotic model membranes
	Tuerkova et al. 2020)	Purchased synthesised lipids	POPC, POPG	Investigate the mechanism of action regarding pore formation induced by kinked helical antimicrobial peptides via fluorescence leakage assays
SUV	Kiss et al. 2021)	Purchased *E. coli* (EH100) LPS extracts and synthesised lipids	Extracted: Ra-LPS Synthesised: DMPC	Facile development of synthetic bacterial membrane models through the step-by-step construction of SUVs
	Brian Chia et al. 2011)	Purchased *E. coli* B (ATCC 11,303) total lipid extract, bovine brain total lipid extract, and synthesised lipids	Extracted: PG, PE, CL, unspecified lipid content Synthesised: DMPC, DMPG	Investigation of peptide selectivity using vesicles to show that natural lipid extracts compare better to MIC values than synthetic lipids
	Bharatiya et al. 2021)	Purchased *B. subtilis* LTA extracts and synthesised lipids	Extracted: LTA Synthesised: DPPG, DPPE, TMCL	Investigate how different compositional variations of LTA alter the structural integrity and stability in model GP membranes
	Bogdanov et al. 2020)	Lipid extracts from *E.coli* strains W3110, W3899, EH150, UE54, BKT12, AL95, AT2033, and *Y. pseudotuberculosis* (O:1b IP32953). Purchased *E. coli* B (ATCC 11,303) polar lipid extracts and purchased plus in-house synthesised lipids	Extracted: PE, PG, CL, PS, Lyso-PE, *N*-acyl-PE, PA, CDP-DAG Synthesised: DPPE, DPPS, TNP-PE, DNP-PE, TNP-LPE, TNP-LPS, TNP-PS, DFDNP-LPE, DFDNP-LPS, DFDNP-PE, DFDNP-PS	To determine how phospholipids are distributed in the IM of GN bacteria and how different phospholipid species influences the distribution and regulation of phospholipid species across the leaflets. The phospholipid asymmetry is discussed in the context of bacterial growth, phospholipid synthesis and translocation, and adjustments in the physical and chemical properties of the membrane
	Cheng et al. 2011)	Purchased bovine heart CL extract and synthesised lipids	Extracted: CL Synthesised: POPG, POPC, POPE	Investigate how the lipid composition in GP and GN bacterial models influence the drug-membrane interactions between various cationic antimicrobial peptides
MLV	D’Errico et al. 2010)	LPS extracts from *B. cenocepacia* ET-12 (LMG 16,656), *B. multivorans* (C1576), *A. tumefaciens* (TT111) and *S. enterica* (minnesota R595). Purchased synthesised lipids	Extracted: R-LPS, S-LPS, Re-LPS Synthesised: DOPE	Characterisation of liposome formation based on initial LPS molecular structure
	Pinheiro et al. 2013)	Purchased synthesised lipids	DMPG, DPPE, DPPG	Investigate the drug-membrane interactions between Rifabutin and bacterial and human membrane models using wide- and small-angle X-ray scattering
	Kumagai et al. 2019)	Extracted LPS from *P. aeruginosa* (PA01) and purchased synthesised lipids	Extracted: LPS Synthesised: POPE, POPG, TOCL, DOTAP	Generate model GN and GP membranes to test the function of newly synthesised antimicrobial peptides. The antimicrobials were tested to assess the drug-membrane interactions and killing efficiency
LUV and GUV	Kahveci et al. 2016)	Purchased bovine heart CL extract and synthesised lipids	Extracted: CL Synthesised: DOPE, DOPG	Analyse the interactions between mammalian and bacterial membrane models and conjugated fluorophores. The models were used to assess fluorophore-lipid binding affinity for the selective cell recognition
SUV and GUV	Malishev et al. 2018)	Purchased bovine heart CL extract and synthesised lipids	Extracted: CL Synthesised: DOPE, DOPG	Investigate the differences in protein-membrane interactions of amyloid protein, TasA, between mimic bacterial and eukaryotic cell membranes
SUV and LUV	Pérez-Peinado et al. 2018)	Purchased *E. coli* B (ATCC 11,303) polar lipid extract and synthesised lipids	Extracted: PE, PG, CL Synthesised: POPC, POPG	Determine the mechanism of action of the antimicrobial peptides, crotalicidin, and its fragment, on the Om of GN bacteria. Liposome models specifically were used to analyse preferential binding and the degree of membrane disruption
Unspecified liposome type	Su et al. 2011)	*E. coli* (WBB06) LPS extract and purchased synthesised lipids	Extracted: Re-LPS Synthesised: POPE, POPG, DEPE	Determination of Gram selectivity among β-hairpin AMPs using LPS-based model systems
	Hancock and Nikaido 1978)	*P. aeruginosa* (PAO1) LPS and lipid extracts, and *S. typhimurium* (LT2M1) LPS and lipid extracts	Unspecified lipid content, R-LPS, S-LPS	Develop an improved method to separate the OM and IM of *P. aeruginosa.* Saccharide retention between liposomes and proteoliposomes was also investigated to compare exclusion limits between *P. aeruginosa* and enteric bacteria*, S. enterica*
	Ruhr and Sahl 1985)	*S. cohnii* (22), *B. subtilis* (W23), *M. luteus* (ATCC 4698) and soybean lipid extracts	Unspecified lipid content, Soy-PC	To determine the effect of the peptide antimicrobial, nisin, on the membrane potential and transport processes of GP bacteria
	Dombach et al. 2020)	Purchased *E. coli* B (ATCC 11,303) polar lipid extract	PE, PG, CL	Investigate the mechanism of action of a small molecule found in macrophages, JD1, that declines the survival and/or growth of GN bacteria

^*^See Supplementary Information (Sect. 1 and 2) for bacterial and lipid species acronym definitions, respectively

Often GUVs or LUVs are used that contain either bacterial lipid extracts (> 4 lipid species), or synthetic lipids determined by the user (< 3 lipid species) asymmetrically arranged in a bilayer. Liposome formation using bacterial lipid extracts provide a more biologically attune system as various lipid species and their native molecular variants are inherently incorporated. Under an artificially user-defined composition, the inner and outer leaflets for GP liposome models commonly contain PG, lysyl-PG, and CL, whilst GN liposome models commonly contain PE, PG, and CL and uncommonly LPS. Liposome models have been utilised to investigate basic structural (lipid domain architecture, rigidity, diffusion, and lateral organisation) and rheological (constriction, shrinkage, and invagination) membrane properties. In addition, protein and peptide-lipid interactions (Saliba et al. 2014; Su et al. 2011), lipid composition-dependent uptake, release, and molecule function (i.e. membrane-targeting antibiotics) (Kilelee et al. 2010; Som and Tew 2008; Brian Chia et al. 2011), pore formation (Samuel and Gillmor 2016; Sborgi et al. 2016), and protein activity (Carrasco-López et al. 2011; Sasaki et al. 2019) have been explored.

Liposome models have been developed for the ESKAPE pathogens and have been used to investigate host–pathogen interactions, membrane permeability, and the effect of membrane composition on antimicrobial susceptibility (Turner et al. 2015; Cheng et al. 2014; Lombardi et al. 2017; Zhang et al. 2014; Hancock and Nikaido 1978; Ciesielski et al. 2013; Lee et al. 1992; Mitchell et al. 2016). Liposomes from synthetic PC and PG lipids and *S. aureus* lipid extracts were used to determine the effects of lipid acyl chain branching on antimicrobial peptide activity (Mitchell et al. 2016). This was achieved by measuring efflux kinetics of the encapsulated fluorescent dye carboxyfluorescein, mediated by the model peptide δ-lysin. Liposomes composed of anteiso-branched isomers were less susceptible to peptide-induced perturbations than liposomes containing iso-branched isomers. In addition, liposomes made from *S. aureus* extracts were more resistant to peptide-induced perturbation than liposomes composed of synthetic lipids, most likely due to the additional increased fraction of anteiso-branched fatty acids.

In a different approach, the association of LPS extracted from *K. pneumoniae* with eukaryotic lipids has been investigated with respect to host immunodetection strategies (Ciesielski et al. 2013). This was achieved by analysing liposome-liposome interactions between pathogen membrane model liposomes containing LPS and PC and host membrane model liposomes containing PC, SL, and cholesterol. LPS preferentially segregated in ordered SL/cholesterol rich domains which was linked to the evolutionary drive for eukaryotic cells to generate, within such domains, a sensory protein for bacterial detection. The permeability of various carbapenems via porins in proteoliposomes reconstituted from lipids extracted from the OM of susceptible and resistant strains *E. cloacae* has also been studied (Lee et al. 1992). Carbapenem permeability and efficacy was highly dependent on the lipophilic constitution of the OM and the amount and type of porins present.

While liposomes are very useful systems to study, they pose some challenges for detailed biophysical studies. Lipid composition is often difficult to control (Rideau et al. 2018; Weinberger et al. 2013). Methods to enhance compositional complexity have been developed (Göpfrich et al. 2019; Pautot et al. 2003); however, they can inhibit surface property analysis (Rideau et al. 2018). The metastable structure of liposomes and their susceptibility to lipophilic, oxidative, and hydrolytic degradation offers poor long-term stability (Akbarzadeh et al. 2013; Nkanga et al. 2019). Additionally, lipids often have relatively high phase transition temperatures which impede liposome formation (Eeman and Deleu 2010; Vestergaard et al, 2008). Finally, despite existing stabilisation methods (Schmid et al. 2015), protein reconstitution in liposomes still remains a challenge (Chan and Boxer 2007; Siontorou et al. 2017).

### Solid-supported bilayers:

**Fig. 3:**
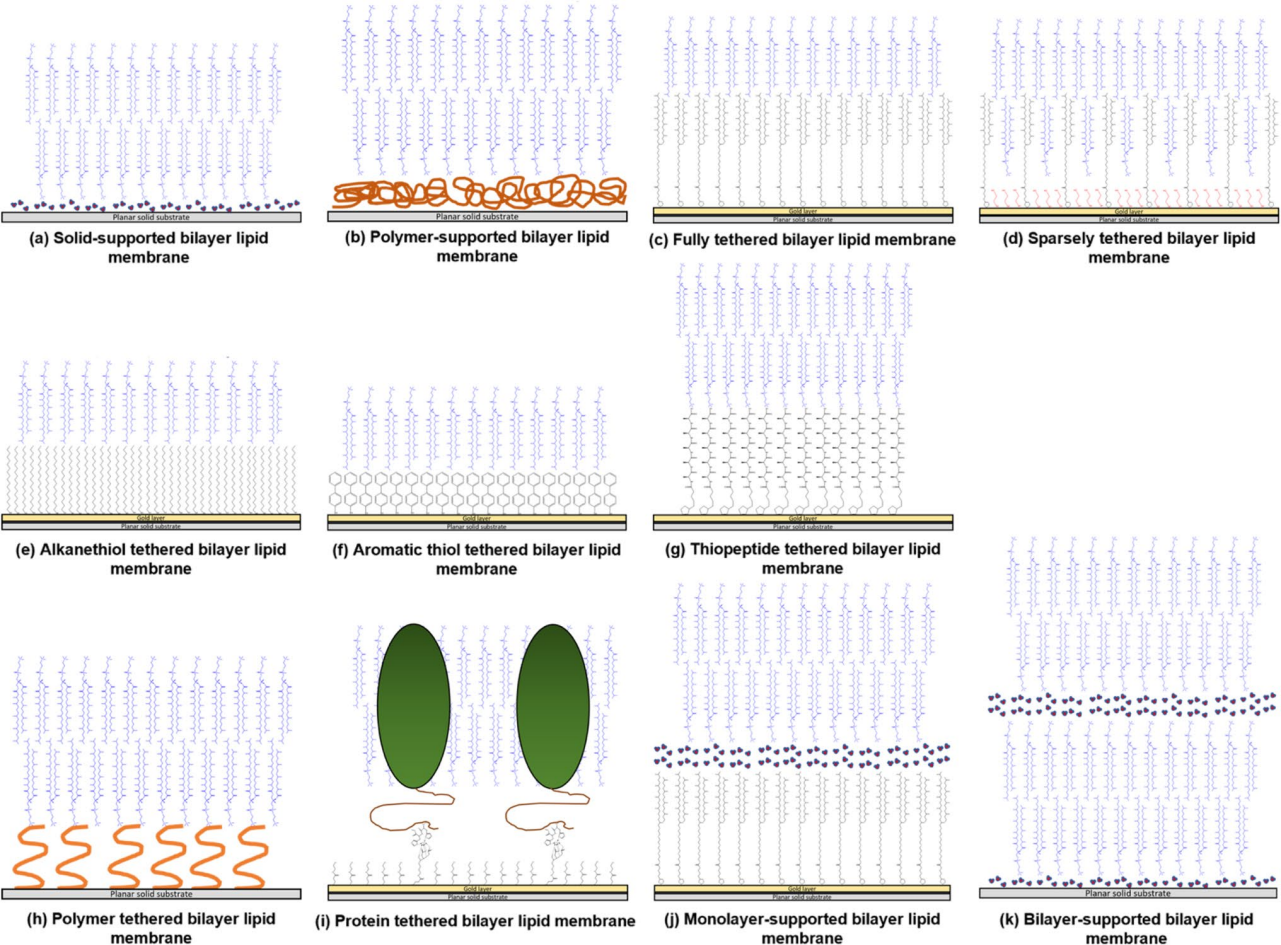
Schematic representation of various solid supported model membrane architectures. Please see text for details

Solid-supported bilayer lipid membranes (sBLMs) consist of a lipid bilayer that is placed onto a solid substrate either via direct contact, via separation by a polymer cushion, or allowed to float directly above a covalently-bound self-assembled monolayer or a supported bilayer (Fig. 3) (Andersson and Köper 2016; Belegrinou et al. 2011; Sackmann 1996; Foglia et al. 2015). Tethered bilayer lipid membranes (tBLMs) are sBLMs with the proximal bilayer leaflet covalently linked to the substrate though thiolipid, oligopeptide, alkane- and aromatic-thiol, polymer, or protein anchors (Andersson and Köper 2016; Andersson et al. 2018b; Jackman et al. 2012; Li et al. 2015; Köper 2007). sBLMs and tBLMs have good electrical sealing properties, are air-stable, and can be formed via Langmuir transfer, vesicle fusion, or solvent-exchange techniques (Andersson et al. 2020; Jackman et al. 2012; Girard-Egrot and Maniti 2021; Kurniawan et al. 2018; Richter et al. 2003).

Gold is the most commonly utilised substrate material for sBLMs and tBLMs due to its stability, facile functionalisation, and versatility in surface analysis techniques (Andersson and Köper 2016). However, other substrates including mercury, quartz, glass, aluminium oxide, indium tin oxide, silicon oxide, sapphire, mica, silver, and titanium oxide can also be utilised (Andersson et al. 2018b; Girard-Egrot and Maniti 2021; Clifton et al. 2020; Giess et al. 2004).

Surface sensitive techniques such as surface plasmon resonance, ellipsometry, neutron or X-ray reflectometry, atomic force microscopy, electrochemical impedance spectroscopy, quartz crystal microbalance with dissipation monitoring, and infrared reflection absorption spectroscopy are well-suited methods of surface analysis for these planar systems in aqueous solution (Ferhan et al. 2017; Wittenberg et al. 2014; Steltenkamp et al. 2006).

While these membrane systems commonly have simple lipid compositions, increased biological accuracy can be achieved in both sBLMs and tBLMs by customising the lipid composition to change membrane electrical sealing and structural properties (Andersson and Köper 2016; Andersson et al. 2018b; Girard-Egrot and Maniti 2021). tBLMs can also change the aforementioned membrane properties and facilitate protein incorporation by customising the tethering type, composition, and density. The OM and IM of various non-pathogenic and pathogenic bacteria have been modelled using both tBLMs and sBLMs (Table 3) (Paulowski et al. 2020; Pérez-Peinado et al. 2018; Weiss et al. 2010; Clifton et al. 2013; Paracini et al. 2018; Hughes et al. 2019; Dodd et al. 2008; Michel et al. 2017; Adhyapak et al. 2020; Nakatani et al. 2019; Hoiles and Krishnamurthy 2015; Schneck et al. 2009; Lee et al. 2020; Nedelkovski et al. 2013; Niu et al. 2017; Sharma et al. 2020; McGillivray et al. 2009).

**Table 3:** Summary of cited solid supported bilayer models, the lipid source, the lipid species utilised and their corresponding research outcomes

Model type	Reference	Lipid source	Lipid species	Research outcomes
tBLM	Andersson et al. 2018a)	Purchased synthesised lipids and *E. coli* (J5) LPS extracts	Extracted: Rc-LPS Synthesised: DPhyPC, d-DPhyPC	Generate a model membrane that mimics the OM of GN bacteria. Structural and electrical properties were investigated with respect to the influence of divalent ions and antibiotics
	Weiss et al. 2010)	Purchased *E. coli* B (ATCC 11,303) polar lipid extracts	PE, PG, CL	Develop an assay to assess the activity of cytochrome *bo*_*3*_ in response to the substrate, ubiquinol-10, in the presence of multiple different inhibitors
	Nakatani et al. 2019)	Purchased *E. coli* B (ATCC 11,303) polar lipid extracts	PE, PG, CL	Develop a model bacterial architecture to analyse the catalytic behaviour of Type-II NADH:quinone oxidoreductase in the presence of various the substrates (quinone, quinone analogues and NADH) and inhibitors (phenothiazines)
	Hoiles and Krishnamurthy 2015)	Purchased synthesised lipids	POPG, Ether-DPhyPC, DPGE	Investigate pore formation dynamics and reaction-mechanism of the antimicrobial peptide, peptidyl-glycine leucine-carboxyamide, in archaebacterial model membranes
	Nedelkovski et al. 2013)	Purchased synthesised lipids	DPhyPC	Generate a biomimetic bacterial membrane architecture that produces enhanced infrared signals to better analyse the photoexcitation mechanism of photosynthetic reaction centres in *R. sphaeroides*
	Niu et al. 2017)	Purchased synthesised lipids and LPS extract from *S. enterica* (minnesota R595)	Extracted: Lipid A Synthesised: DPhyPC, DPhyPG	Investigate the molecular mechanism, interactions, and impact of the antimicrobial peptide, V4, on the electrical and mechanical properties of bacterial membrane models
	McGillivray et al. 2009)	Purchased synthesised lipids	DPhyPC	Develop a model bacterial membrane to analyse the structural and electrical properties and lipid-protein interactions of *α*-hemolysin channels derived from *S. aureus*
	Hsia et al. 2016)	Purchased synthesised lipids and *E. coli* (JC8031) lipid extracts	Extracted: unspecified lipid content from the extracts Synthesised: DOPC, PEG5000-PE	Develop a model membrane of the OM of GN bacteria. The formation of the membrane was characterised kinetically and acoustically to assess surface coverage, vesicle rupture and architecture mass. Properties including membrane diffusivity, mobility, viscoelasticity and lipid and protein symmetry were also investigated. Changes in membrane properties, mass and kinetics were also investigated in the presence of antibiotics
	Thomas et al. 1999)	Purchased synthesised lipids and *E. coli* (K12 D31m4) lipid A extracts	Extracted: DPLA Synthesised: DMPC, Biotin-PE	Investigate and identify the sequestering effectiveness and neutralisation mechanism between LPS and polymyxin B compared to polymyxin B synthetic peptide mimics
	Spencelayh et al. 2006)	*E. coli* JM1100 (pPER3) and purchased egg lipid extracts	Egg-PC, unspecified lipid content from the *E. coli* extracts	Generate a biomimetic bacterial membrane that facilitates the in vitro synthesis of peptidoglycan using native precursors. The binding behaviour between different antibiotics and the peptidoglycan precursors
	Mirandela et al. 2019)	Purchased synthesised lipids and *E. coli* B (ATCC 11,303) polar lipid extracts	Extracted: PG, PE, CL Synthesised: POPC	Investigate how the lipid-protein interaction between a mimetic GN lipid bilayer and an ammonium transporter protein native to *E. coli* affects transporter activity
	Maccarini et al. 2017)	Purchased synthesised lipids	DMPC, GDPE, DPEPC, DOPC, DOPE, DMPA, cholesterol	Develop a procedure to optimise the cell-free production of and incorporation of a porin from *P. aeruginosa* in a functional conformation
	Jeuken et al. 2006)	Purchased *E. coli* B (ATCC 11,303) polar lipid extracts	PG, PE, CL	Characterise the function and structure of redox-active enzyme, cytochrome bo_3_, derived from *E. coli*
	Jeuken et al. 2005)	Purchased *E. coli* B (ATCC 11,303) polar lipid extracts and egg lipid extracts. *B. subtilis* (3G18/pBSD1200) lipid extracts	PG, PE, CL, Lysine-Acyl-PG, egg-PC, unspecified lipid content from the *B. subtilis* extracts	Electrochemically characterise the function of redox-active membrane protein, succinate menaquinone oxidoreductase, native to *B. subtilis*
	Dupuy et al. 2018)	Purchased synthesised lipids and *E. coli* (O111:B4) LPS extracts	Extracted: S-LPS Synthesised: POPE, POPG, TOCL, POPC, DOTAP, KDO2, DLPG	Develop model GP and GN bacterial membranes to elude the biophysical interaction mechanism between the antimicrobial peptide Colistin and different lipid compositions
	Hughes et al. 2019)	Purchased synthesised lipids and *E. coli* (EH100) LPS extracts	Extracted: Ra-LPS Synthesised: d-DPPC	Collect biophysical information and investigate the physical properties of the OM of GN bacteria using model membranes and computational simulations
	Mohamed et al. 2021)	Lipid extracts *from P. aeruginosa* (PA14), *A. baumannii* (LAC-4) and *E. cloacae* (ATCC 13,407) and purchased synthesised lipids	Extracted: unspecified lipid content but LPS was detected and quantified Synthesised: PEG5000-POPC, PEG5000-DHPE	Generate OM model bilayers of three GN ESKAPE pathogens and investigate the model’s biophysical characteristics, and drug-membrane interactions with various antimicrobial compounds
sBLM	Adhyapak et al. 2020)	*M. smegmatis* (mc^2^155) lipid extracts	PA, PE, PG and PI (including lyso forms); CL; DAG (including meromycolyl forms); SfL; DAT; GPepL; MA (including alpha and keto forms); PIM (including mono-acylated forms); TAT; MG; MPM; TDM; MB (including carboxy, cell-bound iron-loaded, monodeoxy, dideoxy and hybrid forms); MQ; PDIM; Ac2SGL; TG; DG; PCA (including hydroxy forms); CET; GPD; MCA; MPanA; MpenA; MSA; MCSA; L5P	Investigate the membrane lipid domain architecture, fluidity, packing, dynamics, synthesis regulation and lateral organisation in protein-free membrane models of mycobacteria
	Schneck et al. 2009)	*S. enterica* (R60 and R595) lipid extracts	Lipid A, Ra-LPS-Ra, Re-LPS	Model the influences of different LPS mutations on the mechanical properties and intermembrane interactions in the presence and absence of divalent ions using GN bacterial OM models
	Lee et al. 2020)	*E. coli* BL21 (K-12 MG1655) total lipid extracts	PE, PG, PA	Investigate the impact of the antimicrobial peptide, maculatin 1.1, on the mechanical properties of lipid domains in bacterial membrane models simulating exponential and stationary growth phases
	Sharma et al. 2020)	Purchased *E. coli* B (ATCC 11,303) total lipid extracts and *E. coli* (O111:B4) LPS extracts and synthesised lipids	Extracted: S-LPS, PE, CL, PG, unspecified lipid species Synthesised: POPE, ATTO488-DMPE, ATTO647N-DMPE	Generate a model membrane that mimics the OM and IM of *E. coli*. Membrane lipid diffusiveness, fluidity, packing, and mobility was analysed with respect to the transport of the antimicrobial thymol
	Clifton et al. 2015)	Purchased *E. coli* (EH100) LPS extracts and synthesised lipids	Extracted: Ra-LPS, Synthesised: DPPC, d-DPPC	Generate an asymmetric model membrane that mimics the IM and OM of *E. coli*
	Li and Smith 2019)	Purchased synthesised lipids	POPG, DOTAP, TOCL, POPE, TopFluor-PE, TopFluor-TOCL	Develop model GP and GN asymmetric bacterial IMs. Lipid diffusion dynamics was investigated in the presence and absence of antimicrobial peptide binding
	Michel et al. 2017)	Purchased synthesised lipids and LPS extract from *S. enterica* (minnesota R595)	Extracted: Re-LPS Synthesised: SOPE, SOPG, TOCL, d-POPG, d-POPE	Develop and characterise a model GN asymmetrical bacterial IMs to antimicrobial plasticins
	Paulowski et al. 2020)	LPS extracts from *P. mirabilis* (R_45_). Purchased *E. coli* lipid extracts and synthesised lipids	Extracted: PE, PG, R-LPS Synthesised: CL, Rhod-DHPE, NBD-PE, FITC-PE	Demonstrate experimental methods to model the asymmetry of GN bacteria. The model’s usability was assessed for lipid domain analysis and peptide and protein interaction by characterising lipid flip-flop and phase behaviour
	Dodd et al. 2008)	Purchased synthesised lipids and E. coli (BL21(DE3)) lipid extracts	Extracted: unspecified lipid content Synthesised: Egg-PC, TRF-DHPE, NBD-PC	Generate sBLMs that contain mixtures of native E. coli lipids with Egg-PC with the intention of generating a simple model membrane for the study of drug-membrane interactions and numerous process that occur in bacterial membranes. The structural properties of the generated sBLMs were assessed using various surface sensitive analytical techniques
	Clifton et al. 2013)	Lipid A and LPS extracts from E. coli strains F583, EH100 and J5. Purchased synthesised lipids	Extracted: lipid A, Ra-LPS, Rc-LPS Synthesised: DPPC, d-DPPC	Develop a facile two-step approach to modelling the OM of GN bacteria. Via neutron reflectometry, the lipid distribution and coverage between leaflets, and membrane stability and structure were analysed
	Pérez-Peinado et al. 2018)	Purchased *E. coli* B (ATCC 11,303) polar lipid extract and synthesised lipids	Extracted: PE, PG, CL Synthesised: POPC, POPG	Determine the mechanism of action of the antimicrobial peptides, crotalicidin and its fragment, on the Om of GN bacteria. sBLM models specifically were used to analyse the membrane permeabilisation mechanism
sBLM and tBLM	Chilambi et al. 2018)	Purchased synthesised lipids and *E. faecalis* OG1RF (wild type), EFC3C and EFC3Py (resistant strains) extracts	Extracted: unspecified lipid species from extracts, various FAs Synthesised: DPDEPC, GPDE, DOPC, POPG	Investigate the antimicrobial mechanism of antimicrobial conjugated oligoelectrolytes through changes in the fatty acid, genetic and uptake profiles between wild type and resistant strains of *E*. *faecalis*
	Paracini et al. 2018)	Purchased synthesised lipids and LPS extract from E*. coli* (EH100)	Extracted: Ra-LPS Synthesised: d-DPPC	Investigate how the physical structure of the lipid OM of GN bacteria influences the drug-membrane interactions of polymyxin B

^*^See Supplementary Information (Sect. 1 and 2) for bacterial and lipid species acronym definitions, respectively

These architectures often contain a limited number (1–4) of synthetic lipid species; however, they can also contain bacterial lipid extracts (> 4 lipid species) asymmetrically arranged in a bilayer. Unlike user-defined systems which are limited to the number and type of lipid species and their associated molecular variations incorporated, architectures formed from bacterial lipid extracts generate increasingly accurate biological models as various lipid species and their native molecular variants are inherently incorporated. Under user-defined compositions, the inner and outer leaflets of architectures modelling GN and GP bacteria commonly contain one molecular variation of PC. Few architectures have been developed where the inner and outer leaflets contain the most common lipid species or analogues thereof for GN (PE, PG and CL) and GP (PG, CL, and lysyl-PG) bacteria. For sBLM and tBLM systems, lysyl-PG is often substituted with DOTAP as it is more affordable for the increased quantities required to generate the architectures (Dupuy et al. 2018; Li and Smith 2019). Few architectures modelling the membrane of GN or GP bacteria have also been developed to contain LPS (Andersson et al. 2018a; Clifton et al. 2015; Hsia et al. 2016; Thomas et al. 1999) or murein (Spencelayh et al. 2006). The model architectures have been utilised to investigate general structural (thickness, roughness, and lipid density) and electrical membrane properties. In addition, the mechanism of interaction between antibiotic compounds and membrane constituents (Chilambi et al. 2018; Dupuy et al. 2018; Li and Smith 2019), lipid-protein interactions (Mirandela et al. 2019), ion transport (Maccarini et al. 2017), and redox-active enzyme function and characterisation (Jeuken et al. 2006, 2005) have been explored.

Limited architectures have been generated to model the ESKAPE pathogens and investigate electrochemical and structural changes with lipophilic composition (Jiang et al. 2019b; Mohamed et al. 2021; Zang et al. 2021). Recently, a tBLM for *A. baumannii* has been developed to model the OM in the presence and absence of exogenously incorporated omega-3 polyunsaturated fatty acid (PUFA) and docosahexaenoic acid (DHA) (Zang et al. 2021). Both tBLMs generated were asymmetrical and were constructed from lipid samples extracted from *A. baumannii* actively growing in the presence or absence of DHA. The tBLMs were used to determine whether DHA incorporation disrupted the function of efflux system AdeB due to impaired proton motive force retention from induced ion leakage. Both tBLM models were electrochemically similar therefore suggesting that AdeB dysfunction was not due to the membrane’s ability to maintain a proton motive force upon DHA incorporation. sBLM models for *S. aureus* have been developed to assess how upregulation in CL biosynthesis in daptomycin-resistant strains decreases antibiotic susceptibility (Jiang et al. 2019b). PG, lysyl-PG and CL in different concentration ratios were used to mimic resistant and susceptible strains. The daptomycin-resistant strain membrane was found to be thicker than the susceptible strain. The structural changes resulted in concentration-dependent changes in daptomycin interaction. At low daptomycin concentrations, the susceptible strain exhibited decreases in lipid volume whilst high concentrations induced considerable membrane penetration and disruption. In contrast, the resistant-strain exhibited only slight lipid volume reductions for all daptomycin concentrations analysed. This demonstrated that lipid-induced structural modifications can impair daptomycin efficacy.

Both sBLM and tBLM systems possess limitations unique to each architecture. sBLM systems can be unstable due to no linkage between the lipid bilayer and the substrate (Andersson and Köper 2016; Andersson et al. 2018b; Girard-Egrot and Maniti 2021). As a result, measurements requiring days or weeks are difficult to achieve. Direct bilayer-substrate contact can also create an insufficient amount of space for bilayer-spanning protein incorporation (Castellana and Cremer 2006; Andersson and Köper 2016; Alghalayini et al. 2019; Tamm and McConnell 1985). Protein-substrate contact induces denaturation or impaired function which hinders functional, electrical, or structural studies (Alghalayini et al. 2019; Tanaka and Sackmann 2005). Membrane structural and electrical properties are also subject to substrate topology, whereby any substrate imperfections will cause defects in the bilayer and hinder its resistance towards current transfer (Andersson and Köper 2016; Andersson et al. 2018b; Girard-Egrot and Maniti 2021). Using a polymer cushion to support the bilayer can partially reduce substrate topological effects, maintain bilayer fluidity, and prevent substrate-protein contact (Andersson and Köper 2016; Andersson et al. 2018b; Belegrinou et al. 2011). However, polymer cushion swelling behaviour, assembly, thickness, and morphology are difficult to control which dampens the electrical qualities of the lipid bilayer (Naumann et al. 2001, 2002). tBLMs were generated to circumvent all aforementioned limitations of sBLMs. However, the disadvantage of increased stability and electrical sealing in tBLM systems is decreased lateral lipid mobility (Andersson et al. 2018b). Depending upon the application, there are also disadvantages to using different types of tethers (Jackman et al. 2012). Similarly to liposomes, consideration of the lipid phase transition temperature can be crucial to successful lipid incorporation and architecture formation (Eeman and Deleu 2010; Vestergaard and d., Hamada, T., Takagi, M., 2008).

### Computational modelling

**Fig. 4:**
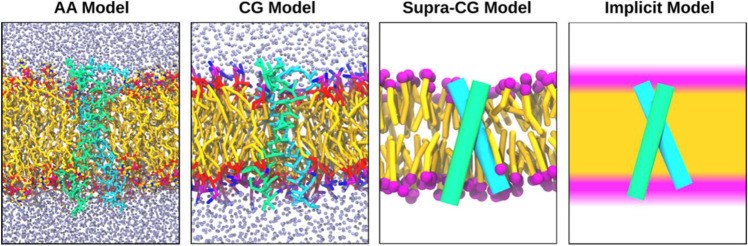
Schematic representation of different resolutions in molecular dynamics simulations of lipid membranes. All atom (AA) resolution explicitly considers all atoms. Coarse-grain (CG) resolution considers small atom groups and their associated hydrogens. Supra-CG resolution represents solvents implicitly and proteins and lipids as qualitative few-bead models. Implicit resolution further integrates out lipid molecules. (modified with permission from (Marrink et al. 2019))

Despite the progress made in developing sophisticated experimental techniques that can directly investigate live bacterial cells and reveal complex lateral membrane organisation processes (Deleu et al. 2014; Lyman et al. 2018; Nickels et al. 2015), analysing the molecular details surrounding membrane organisation still proves difficult (Maity et al. 2015; Marrink et al. 2019). Molecular dynamics (MD) techniques can serve as a “computational microscope” whereby interactions between all constituents in the system can be analysed at an atomistic level (Marrink et al. 2019; Ingólfsson et al. 2016). The quality of the set of parameters that dictate particle interaction, known as the force field (FF), is crucial to the success of an MD simulation (MacKerell 2004). In biomolecular simulations, numerous FFs have been employed: implicit, supra-coarse-grain, coarse-grain, and all-atom (Marrink et al. 2019; Mori et al. 2016). All FFs are similar regarding their main approximations and function; however, the level of resolution between each is distinctive (Fig. 4) (MacKerell 2004). The highest level of resolution is full atomistic detail which is the most commonly utilised model for complex membrane systems. These include bacterial membranes, organelle membranes, plasma membranes and viral envelopes, protein folding, drug-membrane interactions, protein–ligand complex stability, protein–protein interaction modulators, lipid domain formation and behaviour, membrane curvature sensing and formation, membrane remodelling events, and lipid-protein binding site identification and binding strength (Matamoros-Recio et al. 2021; Bennett and Tieleman 2013; Chan et al. 2015; Kabedev et al. 2021; Khan et al. 2019; Lazim et al. 2020; Liu et al. 2021; Parkin et al. 2015; Reddy and Sansom 2016; Singharoy and Schulten 2017). Full atomistic detail significantly expands the predictive power of molecular dynamics simulations. To enhance the spatiotemporal range of MD simulations and decrease system complexity, the lower resolution level FFs can be utilised (Mori et al. 2016; Liu et al. 2021).

Several MD models simulating the OM and IM of bacteria have been constructed at both the atomistic and coarse-grained levels of resolution (Table 4). (Bogdanov et al. 2020; Tuerkova et al. 2020; Hughes et al. 2019; Balusek and Gumbart 2016; Baltoumas et al. 2019; Gao et al. 2020; Kholina et al. 2020; Li and Guo 2013; Abellón-Ruiz et al. 2017; Berglund et al. 2015; Hsu et al. 2017a, 2017b; Ma et al. 2017a, 2017b,^ 2015^; Mehmood et al. 2016; Orekhov et al. 2018; Shearer et al. 2019; Shearer and Khalid 2018; Rice and Wereszczynski 2018; Patel et al. 2016; Piggot et al. 2011; Carpenter et al. 2016; Fleming et al. 2016; Wu et al. 2013, 2014a; Duay et al. 2019; Khondker et al. 2019; Pandit and Klauda 2012; Pothula et al. 2016; Shahane et al. 2019).

**Table 4:** Summary of cited computational models, the bacterial species modelled, and the lipid species utilised and their corresponding research outcomes

Model type	Reference	Modelled bacterial species	Lipid species	Research outcomes
Atomistic (all-atom)	Balusek and Gumbart 2016)	_	POPE, Ra-LPS modelled from E. coli strain K-12	Investigate transport protein-LPS interactions and its effect on Ca^2+^ binding for vitamin transport in GN bacteria
	Duay et al. 2019)	_	POPE, POPG	Determine how Zn ions and pH affect the binding of the antimicrobial peptide, ClavA, to a membrane
	Khondker et al. 2019)	_	POPC, POPS, DMPS	Investigate how the molecular density of a bilayer plays a significant role in the interactions of antimicrobial drugs with the membrane
	Pandit and Klauda 2012)	*E. coli*	POPE, POPG, PMPE, multiple molecular variations of PE and PG that mimic the main constituents of *E. coli* strains K12 LM 3118 and K12 NBRC 3301, PDSPE, PDSPC	Introduce cyclic moieties into the membrane to obtain a more realistic model
	Shahane et al. 2019)	_	POPE, POPG	Determine how membrane composition influences the interaction with various antimicrobial peptides
	Khakbaz and Klauda 2015)	*E. coli*	POPE, PPPE, OSPE, PMPE, QMPE, PSPG, PMPG	Simulated parameters of complex membrane composition and compared how they differ significantly from simpler models
	Lim and Klauda 2011)	*C. trachomatis*	DPhyPC, 13-MpPPC, 14-MpPPC, DPPC, DMPE, DOPE, DOPG, SLPC, PPPE, DSPE, DLPE, POPE, cholesterol	Determine how increased lipid chain branching affects bilayer properties such as elastic modulus and chain order
	Jin et al. 2021)	_	DOPC, POPE, POPG	Demonstrates the interaction of model membranes with various native and non-native small molecules used in quorum sensing
	Lee et al. 2017)	*P. aeruginosa, E. coli*	PPPE, PVPG, PVCL, R-LPS, S-LPS	Investigate how the composition of a membrane influences its interaction with an OM protein
	Ocampo-Ibáñez et al. 2020)	*P. aeruginosa and K. pneumoniae*	POPE, PMCL, POPG	Investigates how the interactions between the membrane and the cationic antimicrobial peptide, CecD, depends on the membrane composition
	Alkhalifa et al. 2020)	*E. coli, S. aureus*	POPC, DLPG, DLPE, TMCL	Determines how the membrane composition influences membrane interaction with various quaternary ammonium compounds
	Piggot et al. 2011)	*E. coli, S. aureus*	LPS, Lysyl-DPPG, POPE, POPG, DMPG, DPPE, CL	Demonstrates how membranes of various lipid composition show different electroporation properties
	Lins and Straatsma 2001)	*P. aeruginosa (PAO1)*	PE, R-LPS	Detailed description of the construction of an LPS membrane
	Yu and Klauda 2018)	*P. aeruginosa (PAO1)*	POPE, POPG, YOPE, PMSPG, PMSPE, DPPE, YOPG, DPPG	Description of a simulation using the CHARMM (Chemistry at Harvard Macromolecular Mechanics) FF to simulate in IM of *P. aeruginosa*
	Hwang et al. 2018)	*E. coli*	POPE, POPG, PMPE, QMPE, PMPG, PSPG, OSPE, Ra-LPS	Mechanical properties of the membrane are influenced by both the cell wall as well as the OM
	Bogdanov et al. 2020)	*_*	DOPE, DOPG, TOCL, FDNB-PE	Elucidate the mechanism behind the inability of 1,5-difluoro-2,4-dinitobenzene to be able to cross-link PE based on phospholipid location in GN bacterial model membranes
	Piggot et al. 2013)	*E. coli*	POPC, PVPE, PVPG, PVCL, Rd-LPS	Model a transporter protein FecA, native to *E. coli* to identify various LPS-protein interactions and determine how it affects the conformational dynamics of FecA
	Kirschner et al. 2012)	*P. aeruginosa (PAO1)*	R-LPS, DPPE	Extend the GLYCAM06 FF to incorporate a new set of parameters that expands the number of monosaccharides that can be added to LPS and, consequently, improve the structure reproduction and membrane permeability for GN bacterial membrane models
	Wu et al. 2013)	*E. coli*	R-LPS, S-LPS	To build and model each LPS constituent based on chemical and spectroscopy investigations. Each consistent in LPS was used to gain insight on LPS properties, LPS molecule dynamics and LPS structure within an LPS bilayer. The addition of the O-antigen was also implemented to investigate how the O-antigen chain heterogeneity influenced membrane dynamins, structure, and properties. Simulations of the O-antigen were validated via NMR
	Dias et al. 2014)	*P. aeruginosa*	DPPE, R-LPS	Investigate how the chemical remodelling of LPS affects the electrostatic properties and structural dynamics of the OM of GN pathogen *P. aeruginosa*
	Wu et al. 2014a)	*E. coli*	PPPE, PVCL, PVPG, R-LPS	Investigate the structural properties the E. coli OM and any protein-lipid interactions experienced between the OM and phospholipase A
	Carpenter et al. 2016)	*E. coli*	Re-LPS, PE, PG, CL	Determine the free energy of permeation of ethane, benzene, hexane, ethanol, water, and acetic acid through an OM model of *E. coli*
	Fleming et al. 2016)	*E. coli*	R-LPS, PPPE, PVPG, PVCL	Investigate the conformation flexibility of transmembrane transporter protein, BamA, to determine how membrane interactions with the polypeptide transport-associated domain influence conformation dynamics
	Patel et al. 2016)	*E. coli*	PPPE, PVPG, PVCL, DMPC, R-LPS and S-LPS modelled from the LPS structure of *E. coli* strain K12	Investigate the impact of how structural differences in various LPS molecules affect the function, dynamics, and structure of the transport protein OmpF. In addition, the importance of protein-LPS interactions was investigated to determine ion permeability and pore access behaviour in different LPS environments
	Rice and Wereszczynski 2018)	*S. enterica*	POPE, LPS (8 different variations both modified and unmodified)	Generate symmetric GN bacterial OMs to determine how the key lipid A differences in *S. enterica* alter bacterial virulence via changes in membrane properties
	Hughes et al. 2019)	*_*	DPPC, R-LPS modelled from the LPS structure of *E. coli* strain K12	Investigate the physical properties and biophysical behaviour of the GN bacterial OM including the lateral packing, lipid asymmetry, bilayer density and lipid profile. The results from the simulation were compared to experimental models to determine the degree of agreeability between the methods
	Li and Guo 2013)	*_*	DOPE, DOPG	Investigate drug-membrane interactions to comprehend the mechanism of action of the antimicrobial EO-OPE-1 (C3)
	Gao et al. 2020)	*_*	DPPG, PSPG, PVPG, R-LPS and S-LPS were modelled from the LPS structure of *E. coli*	Determine changes in membrane structural properties and lipid-membrane interactions upon the incorporation of enterobacterial common antigen glycoconjugates
Course-grain	Ma et al. 2017b)	*H. pylori, P. gingivalis, B. fragilis, B. pertussis, C. trachomatis, C. jejuni, N. meningitidis, and S. enterica*	Lipid A variants from each species analysed, DPPE	To investigate how the molecular profile of lipid A significantly affects the biophysical properties of the membrane such as phase transition temperatures
	Ma et al. 2015)	_	R-LPS, S-LPS, DPPE, various polysaccharides	Simulate a full GN bacterial membrane with an OM, peptidoglycan later and an IM
	Oosten and Harroun 2016)	*P. aeruginosa*	R-LPS, POPE	An optimised simulation for a full LPS membrane
	Hsu et al. 2016)	*_*	POPE, Re-LPS, Ra-LPS	Investigate how the interaction of fullerenes with membrane is dependent on the membrane composition, especially the LPS structure
	Shearer et al. 2020)	*E. coli*	POPE, POPG, CL, Re-LPS	To test numerous simulation methods to determine the best protocol for lipid convergence. This is tested by quantifying the potential of mean force for LPS and phospholipid extraction from model GN bacterial IM and OM bilayers, and lateral mixing of LPS and phospholipids within model GN bacterial IM and OM bilayers
	Shearer et al. 2019)	*E. coli*	Re-LPS, Ra-LPS, DPPC, POPG, POPE, S-LPS, S-LPS-PE	To investigate protein-lipid interactions influenced by the amount of LPS, lipid mobility and protein composition on the function of six native proteins in *E. coli*
	Berglund et al. 2015)	*E. coli*	Re-LPS, PVCL, PVPE, PVPG	Investigate the mechanisms of interaction between the antimicrobial peptide, polymyxin B1, with the OM and IM of *E. coli*
	Ma et al. 2017a)	*E. coli*	DPPE, POPG, CL, Lipid A alone, Lipid A attached to its core oligosaccharides	Determine the structural properties of Lipid A with and without its core oligosaccharides, and investigate the stepwise oligomerisation process of OmpF monomers into more complex dimer and trimer structures
	Hsu et al. 2017b)	*_*	Ra-LPS, Re-LPS, POPG, POPE, CL	Generate a new feature for CHARMM-GUI *Martini Maker* via simulating micelle, nanodisc, vesicle, and bilayer systems in the absence and presence of membrane proteins to allow users to model complex bacterial OMs containing LPS
	Orekhov et al. 2018)	*_*	DPPE, POPC, POPE, POPG, Ra-LPS modelled from *P. aeruginosa strain PAO1*	Investigate the solvation behaviour of substituted polycationic metallophthalo-cyanines, which can result in photodynamic inactivation of GN and GP bacteria, in model bacterial membranes. The models were further utilised in investigating the molecular structure of substituted polycationic metallophthalo-cyanines, and their interactions with the membrane
	Mehmood et al. 2016)	*E. coli*	POPE, POPG, CL	Determine which phospholipids specifically bind to the ATP-binding cassette transporter McjD in different phospholipid membrane compositions, and investigate how they impact the function and stability of the transporter
	Shearer and Khalid 2018)	*_*	POPE, POPG, CL, LPS	Investigate the differences in membrane dynamics and structure between symmetrical and asymmetrical GN bacterial membranes in the presence and absence of transmembrane proteins
	Hsu et al. 2017a)	*E. coli (K12)*	POPE, PVPG, CL, Re-LPS	Construct a model IM and OM of E. coli decorated with various native membrane proteins and connected by the transmembrane multi-drug efflux protein complex AcrBZ-ToIC. The model was used to investigate membrane curvature based, lipid diffusion, protein and lipid movement, lipid flow, lipid movement and protein-lipid interactions
	Kholina et al. 2020)	*_*	POPG, POPE	Determine how various cationic antiseptics interact with model membranes by monitoring membrane structural changes
	Tuerkova et al. 2020)	*_*	POPC, POPS, POPG	Determine how kinks in helical antimicrobial peptides affects membrane pore formation
Atomistic (all-atom) and course-grain	Abellón-Ruiz et al. 2017)	*_*	Re-LPS, POPE	Characterise and analyse the functional mechanism, structure, and lipid membrane interactions of the GN OM lipoprotein MlaA
	Baltoumas et al. 2019)	*_*	LPS (modelled from *P. aeruginosa* and *E. coli*), Lipid A (modelled from *E. coli*, *P. aeruginosa*, *H. pylori*, *N. meningitidis*), POPC, DOPC, DSPC, DPPC, DMPC, DLPC, POPE, DOPE, DSPE, DPPE, DMPE, DLPE, POPS, DOPS, DSPS, DPPS, DMPS, DLPS, POPG, DOPG, DSPG, DPPG, DMPG, DLPG, CL (both mono-and di-anionic forms)	Comparing the versatility and abilities of the program GNOMM (Gram-Negative Outer Membrane Modeler) in constructing and analysing the complex OM of GN bacteria across four different FFs

^*^See Supplementary Information (Sect. 1 and 2) for bacterial and lipid species acronym definitions, respectively

These models often contain 2 or more different lipid species asymmetrically arranged in a bilayer, with the outer and inner leaflets composed primarily of LPS (restricted to the outer leaflet) and/or a mixture of PE, PG and sometimes CL. To compensate for the significant variation in the constituents of the phospholipids and LPS between bacterial strains and species, a range of different phospholipid and LPS fragments and variants have been parametrised for use in MD programs (Lee et al. 2018; Wu et al. 2014b). The models have been utilised to characterise and explore various membrane channels and bacterial membrane properties including divalent cation binding, density, diffusion, packing, rigidity, and average area per lipid. In addition, lipid changes between bacterial growth cycles (Khakbaz and Klauda 2015; Lim and Klauda 2011), effects of mechanical and oxidative stressors (Hwang et al. 2018), molecule permeation and partitioning (Jin et al. 2021; Hsu et al. 2016), and the lipophilic influence on membrane protein function and packing (Khalid et al. 2015; Patel et al. 2017) have also been explored.

Bacterial membranes modelling the ESKAPE pathogens have also been simulated to investigate drug-membrane interactions, lipid-protein interactions, and structural changes associated with bacterial pathogenesis (Zang et al. 2021; Piggot et al. 2011; Lee et al. 2017; Ocampo-Ibáñez et al. 2020; Alkhalifa et al. 2020; Lins and Straatsma 2001; Yu and Klauda 2018; Kirschner et al. 2012; Dias et al. 2014; Oosten and Harroun 2016; Chakraborty et al. 2020; Kim et al. 2016). Models for *A. baumannii* containing the OM/IM spanning AdeB RND drug-efflux complex in the presence and absence of incorporated host-derived PUFAs, arachidonic acid, and DHA have been developed within the coarse-grained FF to investigate PUFA-mediated antibiotic susceptibility (Zang et al. 2021). All three simulated membranes were asymmetrical, contained three different lipid species notably PG, CL, and PE and 2–7 molecular variations of each. PUFA incorporation was shown to morphologically disrupt AdeB, resulting in impaired efflux function and presented a potential weakness in *A. baumannii’s* MDR capacity. Chakraborty et al. (2020) also explored various drug-membrane-dependent interactions of two antimicrobial peptides, battacin analogues octapeptide 17 and pentapeptide 30, with the IM of *S. aureus* using an atomistic FF (Chakraborty et al. 2020). The IM was an asymmetric three-component mixture predominately of PG, lysine-PG, DPG, and CL. Kim et al. (2016) modelled homogenous bilayers from 12 pathogenic bacterial species, including *A. baumannii*, *K. pneumoniae*, and *P. aeruginosa*, using an atomistic FF to investigate atomistic-scale similarities and differences in membrane properties induced by the structural variations in LPS (Kim et al. 2016).

Molecular dynamic simulations can provide a detailed picture of membrane structure, yet they sometimes limited by the high complexity of biological membrane systems. For comprehensive reviews of the analytical limitations of MD simulations, see Marrink et al. (2019) (Marrink et al. 2019) and Goossens and Winter (2018). (Goossens and Winter 2018) Developments in the field are however very promising.

## Outlook

The membrane models used to mimic pathogenic bacterial membranes and the techniques used to analyse them have provided useful information on the lateral organisation of these adaptable quasi two-dimensional architectures during resistance development. Each architecture possesses individual advantages and limitations when investigating drug-membrane interactions, lipid-protein interactions, host–pathogen interactions, and structure-induced bacterial pathogenesis. As in vitro modelling systems advance, the quest for increased realism has not ceased. Key challenges include observing and incorporating complex membrane proteins such as drug-efflux proteins, connecting theoretical and experimental results, and incorporating more complex lipophilic assemblies. Current model systems are created utilising well-defined lipid mixtures, and whilst simplification is necessary for specific membrane-mediated interaction analyses, oversimplification provides an insufficient understanding of complex bacterial membrane systems and processes. By incorporating more complex compositions (proteins and lipids), insights into essential pathogen resistance development processes, membrane-targeting antimicrobial mechanisms, and generating fully artificial architectures that safely captures numerous essential pathogenic biological features can be made to help combat the devastating consequences of antibiotic resistance.

## Supplementary Information

Below is the link to the electronic supplementary material.

Supplementary file1 (DOCX 31 KB)
