# Supplementary material for: Model architectures for bacterial membranes
Source: Biophys Rev. 2022 Mar 7;14(1):111–43. doi: 10.1007/s12551-021-00913-7 (PMC8921416; doi:10.1007/s12551-021-00913-7)
Supplement: Supplementary file 1 — Supplementary file1 (DOCX 31 KB) [file 12551_2021_913_MOESM1_ESM.docx]

**Supplementary Information**

Model Architectures for Bacterial Membranes

Ashley B. Carey,^a^ Alex Ashenden^a^ and Ingo Köper^a^

1. Flinders Institute for Nanoscale Science and Technology, College of Science and Engineering, Flinders University, Adelaide, South Australia 5001, Australia.

**1. Bacterial acronyms:**

*Enterococcus faecium = E. faecium*

*Staphylococcus aureus = S. aureus*

*Klebsiella pneumoniae = K. pneumoniae*

*Acinetobacter baumannii = A. baumannii*

*Pseudomonas aeruginosa = P. aeruginosa*

*Enterobacter cloacae* = *E. cloacae*

*Enterobacter hormaechei = E. hormaechei*

*Enterobacter aerogenes = E. aerogenes*

*Escherichia coli = E. coli*

*Saccharomyces cerevisiae = S. cerevisiae*

*Pichia cirerri = P. cirerri*

*Clostridium botulinum = C. botulinum*

*Burkholderia cenocepacia = B. cenocepacia*

*Burkholderia multivorans = B. multivorans*

*Agrobacterium tumefaciens = A. tumefaciens*

*Salmonella enterica = S. enterica*

*Salmonella typhimurium = S. typhimurium*

*Staphylococcus cohnii = S. cohnii*

*Bacillus subtilis = B. subtilis*

*Micrococcus luteus = M. luteus*

*Mycobacterium smegmatis = M. smegmatis*

*Enterococcus faecalis = E. faecalis*

*Chlamydia trachomatis = C. trachomatis*

*Helicobacter pylori = H. pylori*

*Porphyromonas gingivalis = P. gingivalis*

*Bacteroides fragilis = B. fragilis*

*Bordetella pertussis = B. pertussis*

*Campylobacter jejuni = C. jejuni*

*Neisseria meningitidis = N. meningitidis*

*Proteus mirabilis = P. mirabilis*

*Yersinia pseudotuberculosis = Y. pseudotuberculosis*

*Yersinia kristensenii = Y. kristensenii*

*Enterococcus faecium*, *Staphylococcus aureus*, *Klebsiella pneumoniae*, *Acinetobacter baumannii*, *Pseudomonas aeruginosa*, and Enterobacter species = ESKAPE

*Rhodobacter sphaeroides = R. sphaeroides.*

**2. Lipid acronyms:**

1-palmitoyl-2-oleoyl phosphatidylcholine = POPC

1-palmitoyl-2-oleoyl-sn-glycero-3-phospho-(1′rac-glycerol) = POPG

Rhodamine-1,2-dioleoyl-sn-glycero-3- phosphatidylethanolamine = Rhod-DOPE

Smooth lipopolysaccharide = S-LPS

Fluorescein isothiocyanate lipopolysaccharide = FITC-LPS

Rhodamine-1,2-dihexadecanoyl-sn-glycero-3-phosphatidylethanolamine = Rhod-DHPE

Monophosphoryl lipid A = MPLA

1, 2-diphytanoyl-sn-glycero-3-phosphocholine = DPhyPC

Deuterated 1, 2-diphytanoyl-sn-glycero-3-phosphocholine = d-DPhyPC

1, 2-diphytanyl-ether-glycero-phosphatidylcholine = ether-DPhyPC

Diphosphatidylglycerol ether = DPGE

1,2-diphytanoyl-sn-Glycero-3-[Phospho-rac-(1-glycerol)] = DPhyPG

1,2-dioleoyl-sn-glycero- 3-phosphocholine = DOPC

1,2-dioleoyl-sn-glycero-3-phosphatidylethanolamine-N-[methoxy(polyethylene glycol)-5000] = PEG5000-PE

1,2-dimyristoyl-sn-glycero-3-phosphocholine = DMPC

1-oleoyl-2-(12-biotinyl(aminododecanoyl))-sn-glycero-3-phosphatidylethanolamine = biotin-PE

Diphosphoryl lipid A = DPLA

Egg L-α-phosphatidylcholine = Egg-PC

Glycerol diphytanyl ether = GDPE

Diphytanyletherphosphatidylcholine = DPEPC

1,2-dioleoyl-sn-glycero-3-phosphatidylethanolamine = DOPE

1,2-dimyristoyl-sn-glycero-3-phosphatidic acid = DMPA

Lysine acylated phosphatidylglycerol = Lysine-Acyl-PG

Tetraoleoyl cardiolipin = TOCL

1-palmitoyl-2-oleoyl-sn-glycero-3-phosphatidylethanolamine = POPE

1,2-dioleoyl-3-trimeathylammonium-propane = DOTAP

Di[3-deoxy-D-manno-octulosonyl]-lipid A = KDO2

Dilauroylphosphatidylglycerol = DLPG

Diacylglycerols = DAG

Sulfolipids = SfL

Diacyltrehaloses = DAT

Glycopeptidolipids = GPepL

Mycolic acids = MA

Phosphatidylinositol mannosides = PIM

Triacyltrehalose = TAT

Monoradylglycerolipids = MG

Mannosyl-β1-phosphomycoketides = MPM

Trehalose dimycolates = TDM

Mycobactins = MB

Menaquinones = MQ

Phthiocerol dimycocerosates = PDIM

Diacylated sulfoglycolipids = Ac2SGL

Triradylglycerolipids = TG

Diradylglycerolipids = DG

Phthioceranic acid = PCA

Carboxylic exochelins threonine = CET

Glycosylated phthiodiolone dimycocerosate = GPD

Mycocerosic acid = MCA

Mycolipanolic acid = MPanA

Mycolipenic acid = MPenA

Mycosanoic acid = MSA

Mycocersoci acid = MCSA

Lipopentapeptides = L5P

ATTO 488 1,2-dimyristoyl-sn-glycero-3-phosphatidylethanolamine = ATTO488-DMPE

ATTO 647N 1,2-dimyristoyl-sn-glycero-3-phosphatidylethanolamine = ATTO647N-DMPE

Dipalmitoylphosphatidylcholine = DPPC

Deuterated dipalmitoylphosphatidylcholine = d-DPPC

1-palmitoyl-2-(dipyrrometheneboron difluoride)undecanoyl-sn-glycero-3-phosphatidylethanolamine = TopFluor-PE

1,1′,2,2′- tetraoleoyl cardiolipin[4-(dipyrrometheneboron difluoride)- butanoyl] = TopFluor-TOCL

Deuterated 1-palmitoyl-2-oleoyl-sn-glycero-3-phosphatidylethanolamine = d-POPE

Deuterated 1-palmitoyl-2-oleoyl-sn-glycero-3-phospho-(1′rac-glycerol) = d-POPG

1-stearoyl-2-oleoyl-sn-glycero-3-phosphatidylethanolamine = SOPE

1-stearoyl-2-oleoyl-sn-glycero-3-phospho-(1′-rac-glycerol) = SOPG

Diphytanyldietherphosphatydylcholine = DPDEPC

Ergosterol = ES

1,2-di-(9Z-octadecenoyl)-sn-glycero-3-phosphoserine = DOPS

1,2-di-(9Z-octadecenoyl)-sn-glycero-3-phospho-(1'-rac-glycerol) = DOPG

1,2-di-(9Z-octadecenoyl)-sn-glycero-3-phosphate = DOPA

1,2-di-(9Z-octadecenoyl)-sn-glycero-3-phosphoinositol = DOPI

N-(stearoyl)- dihydroceramide = dihydrocer

N-(stearoyl)-ceramide = Cer

N-(octadecanoyl)-4R-hydroxysphinganine = phytocer

1-heptadecanoyl-2-(5Z,8Z,11Z,14Z-eicosatetraenoyl)-sn-glycero-3- phospho-(1'-myo-inositol-4'-phosphate) = PIP

Sphingosine = SO

Phytosphingosine = PHS

Dihydrosphingosine = DHS

1,2-di-(9Z-octadecenoyl)-sn-glycero-3-phosphatidylethanolamine-N- [methoxy(polyethylene glycol)-350] = PEG350-PE

1,2-di-(9Z-octadecenoyl)-sn-glycero-3-phosphatidylethanolamine-N- [methoxy(polyethylene glycol)-2000] = PEG2000-PE

1-(cis-9-Octadecenoyl)-2-[12-[(7-nitro-2-,1,3-benzoxadiazol-4-yl)amino]dodecanoyl]-sn-glycerol-3-phospho-rac-(1-glycerol) = NBD-PG

N-(4,4-Difluoro-5,7-Dimethyl-4-Bora-3a,4a-Diaza-s-Indacene-3-Propionyl)-1,2-Dihexadecanoyl-sn-Glycero-3-phosphatidylethanolamine = BodipyFL-PE

1,2-Dipalmitoyl-sn-glycero-3-phosphoglycerol = DPPG

1,2-Dipalmitoyl-sn-glycero-3-phosphatidylethanolamine = DPPE

Egg-L-α-lysophosphatidylcholine = Egg-lyso-PC

N-(7-nitro-benz-2-oxa-1,3-diazol-4-yl) phosphatidylethanolamine = NBD-PE

N-(7-nitro-benz-2-oxa-1,3-diazol-4-yl) phosphatidylcholine = NBD-PC

Texas Red fluorophore 1,2-dihexadecanoyl-sn-glycero-3-phosphatidylethanolamine = TRF-DHPE

N-(Lissaminerhodamine-Bsulfonyl) phosphatidylethanolamine = Rhod-PE

1-palmitoyl-2-stearoyl-(5-doxyl)-sn-glycero-3-phosphocholine = 5-SLPC

1-palmitoyl-2-stearoyl-(14-doxyl)-sn-glycero-3-phosphocholine = 14-SLPC

1,2-dielaidoyl-sn-glycero-3-pphosphatidylethanolamine = DEPE

Rough-LPS = R-LPS (includes Ra-, Re-, Rd-and Rc-LPS variants)

L-α-Phosphatidylcholine = Soy-PC

1,2-ditetradecanoyl-sn-glycero-3-phospho-L-serine = DMPS

1-palmitoyl-2-cis-9,10- methylene-hexadecanoic-acid-glycero-sn-3- phosphatidylethanolamine = PMPE

1-palmitoyl-2-cis-9,10-methylene-hexadecanoic-acid-glycero-sn-3-phosphoglycerol = PMPG

1-palmitoyl-2-dihydrosterculoyl- sn-glycero-3-phosphatidylethanolamine = PDSPE

1-palmitoyl-2-dihydrosterculoyl-sn-glycero-3-phosphocholine = PDSPC

1-oleoyl-2-palmitoleoyl-sn- glycero-3-phosphatidylethanolamine = OSPE

1-pentadecanoyl-2-cis-9,10- methylene-hexadecanoic-acid-sn-glycero-3-phosphatidylethanolamine = QMPE

1-palmitoyl-2-palmitoleoyl-sn-glycero-3-phosphoglycerol = PSPG

1-palmitoyl-2-palmitoleoyl-sn-glycero-3-phosphatidylethanolamine= PPPE

1-palmitoleoyl-2-oleoyl-*sn*-glycero-3- phosphatidylethanolamine = YOPE

1-palmitoleoyl-2-oleoyl-*sn*-glycero-3-phosphoglycerol = YOPG

1-palmitoyl-2-cis-11,12-methylene-stearic-acid-*sn*-glycero-3-phosphatidylethanolamine = PMSPE

1-palmitoyl-2-cis-11,12-methylene-stearic-acid-*sn*-glycero-3-phosphoglycerol = PMSPG

1-13-methylpentadecanoyl-2-palmitoyl-phosphatidylcholine = 13-MpPPC

1-14-methylpentadecanoyl-2-palmitoyl-phosphatidylcholine = 14-MpPPC

1-stearoyl-2-lineoleoylphosphatidylcholine = SLPC

Dimyristoylphosphatidylethanolamine = DMPE

Distearoylphosphatidylethanolamine = DSPE

Dilineoleoylphosphatidylethanolamine = DLPE

1-palmitoyl-2-vacenoyl-sn-glycero-3-phosphoglycerol = PVPG

1-palmitoyl-2-vacenoyl-sn-glycero-3-phosphatidylethanolamine = PVPE

1,1′-palmitoyl-2,2′-vacenoyl-cardiolipin = PVCL

Lysine-phosphatidylglycerol = Lysine-PG

Lysyl-1,2-dipalmitoyl-sn-glycero-3-phosphatidylglycerol = Lysyl-DPPG

1-palmitoyl-2-myristoyl-1,3-bis(*sn*-3′-phosphatidyl)-*sn*-glycerol = PMCL

1,2,-1′,2′-tetramyristoylcardiolipin = TMCL

Sphingolipid = SL

Lyso-phosphatidylethanolamine = Lyso-PE

Lysyl-phoshatidyglycerol = Lysyl-PG

Glycerolphospho-diglycodiacylglycerol = GP-DGDAG

Phosphatidylglycerol = PG

Cardiolipin = CL

Phosphatidylethanolamine = PE

Phosphatidylcholine = PC

Lyso-phosphatidylcholine = Lyso-PC

Phosphatidylinositol = PI

Phosphatidic acid = PA

Phosphatidylserine = PS

Lyso-phosphatidic acid = Lyso-PA

Glycophospholipid = GPL

Acylated-phosphatidylglycerol = Acyl-PG

Monolysocardiolipins = MLCL

Hydroxylated phosphatidylethanolamine = PE-OH

Hydroxylated cardiolipin = CL-OH

Hydroxylated monolysocardiolipins = MLCL-OH

Alanyl-phosphatidylglycerol = Alanyl-PG

Ornithine lipid = OL

Rhamnolipids = RL

Lipoteichoic Acid = LTA

Fatty Acids = FA

2,3‐di‐O‐phytanyl‐sn‐glycerol‐1‐tetraethylene glycol‐D,L‐α‐lipoic acid ester = DPhyTL

Lipopolysaccharide = LPS

2-dimyristoyl-*sn*-glycero-3-phospho-*rac*-(1-glycerol) = DMPG

Deuterated 2-dimyristoyl-*sn*-glycero-3-phospho-*rac*-(1-glycerol) = d-DMPG

Deuterated 1,2-dimyristoyl-sn-glycero-3-phosphocholine = d-DMPC

Fluorescein isothiocyanate phosphatidylethanolamine = FITC-PE

*N*-acylated-phosphatidylethanolamine = *N*-acyl-PE

Cytidine diphosphate diacylglycerol = CDP-DAG

1,2-Dipalmitoyl-sn-glycero-3-phosphoserine = DPPS

Dinitrophenyl-phosphatidylethanolamine = DNP-PE

Trinitrophenol-phosphatidylethanolamine = TNP-PE

Trinitrophenol-phosphatidylserine = TNP-PS

Trinitrophenol-lyso-phosphatidylserine = TNP-LPS

Trinitrophenol-lyso-phosphatidylethanolamine = TNP-LPE

1,5-difluoro-2,4-dinitobenzene-lyso-phosphatidylethanolamine = DFDNP-LPE

1,5-difluoro-2,4-dinitobenzene-lyso-phosphatidylserine = DFDNP-LPS

1,5-difluoro-2,4-dinitobenzene-phosphatidylethanolamine = DFDNP-PE

1,5-difluoro-2,4-dinitobenzene-phosphatidylserine = DFDNP-PS

1,2-dihexadecanoyl-sn-glycero-3-phosphatidylethanolamine-N-[methoxy(polyethylene glycol)-5000] = PEG5000-DHPE

1-palmitoyl-2-oleoyl phosphatidylcholine-N-[methoxy(polyethylene glycol)-5000] = PEG5000-POPC

Di-lauroyl-phosphatidyl-choline = DLPC

Di-stearoyl-phosphatidyl-choline = DSPC

1-palmitoyl-2-oleyl-phosphatidyl-serine = POPS

Di-stearoyl-phosphatidyl-serine = DSPS

Di-lauroyl-phosphatidyl-serine = DLPS

Di-stearoyl-phosphatidyl-glycerol = DSPG

Diphosphatidylglycerol = DPG
